# Cerebrospinal Fluid Testing in Leptomeningeal Progression of HER2-Negative Breast Cancer Reveals HER2 Positivity, Leading to HER2-Targeted Therapy: A Case Report

**DOI:** 10.7759/cureus.55483

**Published:** 2024-03-04

**Authors:** Seraphina Choi, Daniel Cassidy, Patricia Castillo, Eric A Mellon, Carmen Calfa

**Affiliations:** 1 Radiation Oncology, University of Miami Miller School of Medicine, Miami, USA; 2 Pathology, University of Miami Miller School of Medicine, Miami, USA; 3 Radiology, University of Miami Miller School of Medicine, Miami, USA; 4 Medical Oncology, University of Miami Miller School of Medicine, Miami, USA

**Keywords:** next-generation sequencing (ngs), her2-targeted therapy, leptomeningeal disease, precision oncology, breast cancer

## Abstract

The treatment of breast cancer is largely determined by protein expression assays of estrogen receptor, progesterone receptor, and Her2/neu (HER2) status. These prognostic markers may vary due to tumor heterogeneityor the evolution of prognostic markers throughout the course of treatment. This report presents a case of a patient who initially presented with HER2-negative breast cancer and had rapidly progressed on numerous lines of treatment. An analysis of cerebrospinal fluid via next-generation sequencing and biopsy of metastasis to the liver identified HER2-positive cancer, which allowed for the use of trastuzumab deruxtecan, a HER2-targeted therapy. This led to an excellent clinical response with improvement in performance status and quality of life. This case report demonstrates the importance of continuing to follow a patient’s cancer pathology to open the doors for other opportunities for treatment. Cancer has the potential to evolve and there is a benefit of obtaining rebiopsies to ensure the correct targeted therapies are provided to the patient.

## Introduction

In recent years, breast cancer treatment has been influenced by the practice of precision oncology to provide personalized medicine to patients based on their specific tumor characteristics. The treatment of breast cancer is largely determined by protein expression assays of estrogen receptor (ER), progesterone receptor (PR), and Her2/neu (HER2) status. However, these prognostic markers may vary greatly even within one biopsy due to tumor heterogeneity [1-3]. Furthermore, numerous retrospective studies have shown that prognostic markers may convert during tumor progression [4,5]. Given the possibility that pathology results may be subject to sampling bias and that prognostic markers may evolve during the disease course, it is important to accurately identify prognostic markers to provide the correct targeted therapies.

Here, we present a case of a patient who initially presented with HER2-negative breast cancer and had rapidly progressed on numerous lines of treatment. An analysis of cerebrospinal fluid (CSF) via next-generation sequencing (NGS) and biopsy of metastasis to the liver identified HER2-positive cancer, which allowed for the use of HER2-targeted therapy and markedly improved response to treatment.

## Case presentation

**Figure 1:**
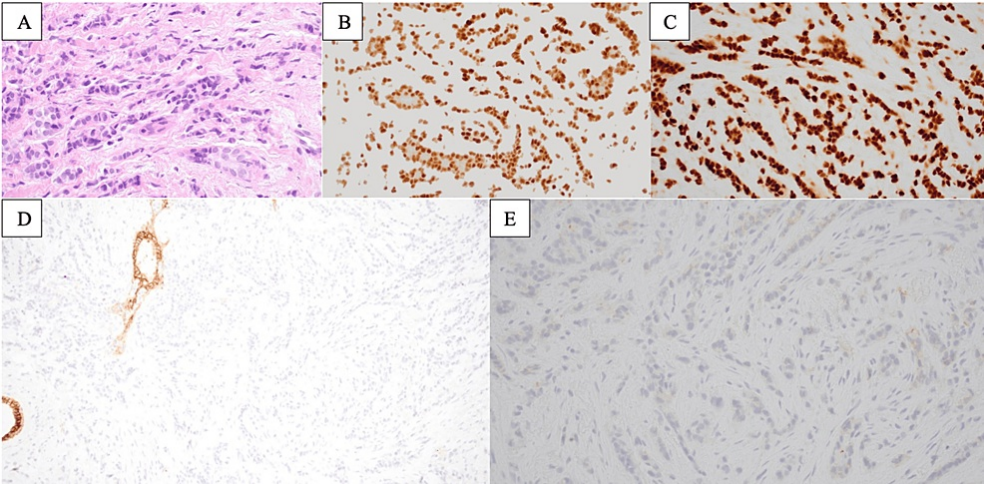
Histologic sections show invasive lobular carcinoma with frequent “single file” growth (A, 400x). Immunohistochemistry for estrogen receptor (B, 400x) and progesterone receptor (C, 400x) are both strongly positive in carcinoma cells. The carcinoma cells are negative for E-cadherin (D, 200x), supporting their lobular phenotype, and negative (1+) for HER2 (E, 400x).

A 54-year-old female presented with a one-year history of back pain as well as new-onset left breast tenderness and nipple retraction in October 2017. Physical exam was remarkable for a 4-cm palpable, ill-defined, mobile mass with nipple retraction in the left breast and a palpable mobile axillary lymph node. A bilateral diagnostic mammogram did not reveal any suspicious mass but did indicate a slight deformity of the nipple-areola complex. Although the initial mammogram did not reveal any discrete masses, this was discordant with initial physical exam findings. This led to a recommendation to pursue additional imaging to further characterize this patient’s breast mass via a diagnostic ultrasound, which showed a lesion measuring 1 x 1.2 cm in the retroareolar region. Left breast ultrasound-guided biopsy results demonstrated invasive lobular carcinoma, grade 2, moderately differentiated, ER strong positivity of 90%, PR strong positivity of 95%, HER2 negativity (1+), and low Ki-67 of 10% (Figure 1). PET/CT scan showed diffusely increased uptake in the bones and lymph nodes. A left iliac crest bone lesion biopsy confirmed the presence of metastatic disease, confirming a diagnosis of de novo stage 4 breast cancer (cT2, cN1, cM1, G2). MyRisk 28-gene panel was negative for any clinically significant mutations, including BRCA1 and BRCA2. MSK-IMPACT® testing detected mutations in PIK3CA, AKT3, CDH1, MAP3K1, and NOTCH4. The patient had a family history of a paternal aunt who was diagnosed with breast cancer at 45 years old, an unspecified cousin who was diagnosed with breast cancer (BRCA1 mutation, del exons 13-24 heterozygous), and a paternal cousin diagnosed with ovarian cancer at an unspecified age. At the time of diagnosis, the patient’s initial Karnofsky performance status (KPS) was 90.

The patient was initially started on letrozole 2.5 mg daily in 2017. After three years on letrozole, disease progression was seen on PET/CT. The patient was switched to fulvestrant 500 mg injection and palbociclib (100 mg daily for three weeks and off for one week) for five months. Due to definitive progression of bone and lymph node metastases, the patient began third-line treatment with enrollment in the phase II trial of radium-223 dichloride in combination with paclitaxel open-labeled clinical trial (NCT04090398) and randomization to paclitaxel single agent. After two months, imaging continued to show disease progression and the patient discontinued participation in the clinical trial. In March 2021, Guardant360® liquid biopsy from blood identified an alteration/biomarker in PIK3CA E542K and CDH1 L725fs. A variant of uncertain significance was identified in TP53 E224D and a synonymous alteration was identified in MAPK1 I56I. The detected alteration in PIK3CA allowed her to begin fourth-line treatment on alpelisib 250 mg daily and tamoxifen 20 mg daily for six months.

**Figure 2:**
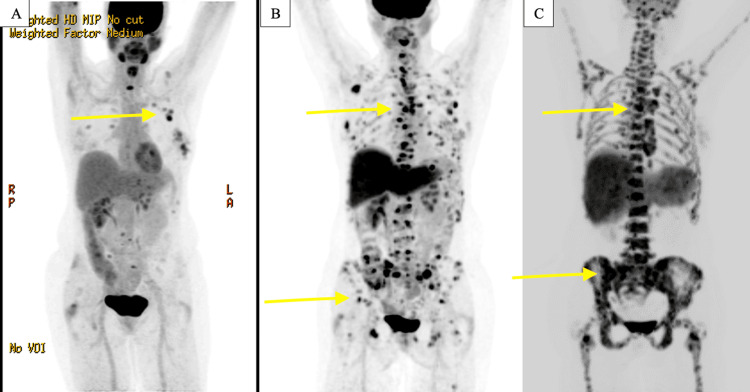
PET/CT scans performed at different points during the evolution of the disease. (A) In December 2020, there was increased fluorodeoxyglucose (FDG) activity in the left breast, axillary lymph nodes, and spine. (B) In June 2021, there was a worsening of metabolic activity in the thoracolumbar spine and pelvic bones. (C) In February 2022, there was further progression given the presence of multiple mediastinal and abdominal lymphadenopathy as well as extensive metastasis in the axial and appendicular skeleton.

**Figure 3:**
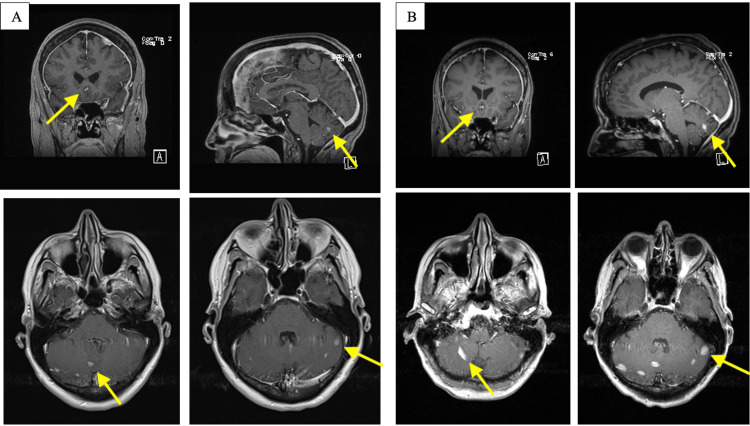
(A) Brain MRI obtained in January 2022 demonstrates pachymeningeal enhancement with a focal dural-based lesion in the left frontal lobe as well as multiple enhancing lesions in the posterior fossa. (B) Follow-up MRI obtained in March 2022 shows an increase in the size and number of brain lesions in the posterior fossa as well as worsening of the meningeal enhancement.

After a C2 lesion was seen on PET/CT (Figure 2), the patient had an MRI of the cervical spine, which was remarkable for widespread osseous metastatic disease. This prompted an MRI of the brain, which showed a left subdural collection with diffuse pachymeningeal enhancement, numerous enhancing lesions within the posterior fossa, and multiple foci along the periphery of the cerebellum, which strongly indicated the presence of diffuse leptomeningeal disease (Figure 3). In January 2022, a lumbar puncture was performed and cytology did not detect malignant cells in the CSF. At the time, the patient was recommended and received conventional whole-brain radiation therapy (WBRT) with 30 Gy (10 fractions). However, the CSF sample was also sent for Biocept testing for next-generation sequencing (NGS), which resulted in the detection of five malignant cells with one cell having HER2 amplification.

**Figure 4:**
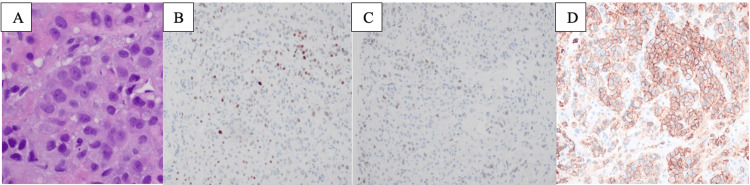
Histologic sections of liver biopsy performed in February 2022 showed involvement by carcinoma with pleomorphic features (A, 600x). The carcinoma cells showed low positivity (subset, weak) for the estrogen receptor (B, 400x) and low positivity for the progesterone receptor (C, 400x) by immunohistochemistry. HER2 immunohistochemistry was shown to be positive (3+) (D, 400x).

After radiation, PET/CT showed a diffuse hypodense appearance of the liver likely representing metastases. The patient was started on capecitabine while liver biopsy was recommended and performed in February 2022. Interestingly, pathology of the biopsy showed metastatic breast carcinoma (with pleomorphic lobular features), negative E-cadherin, positive GATA3, positive TRPS1 with ER weak positivity of 11-50%, PR weak positivity of 11-50%, and HER2 positivity (3+) (Figure 4). Due to the new finding of HER2 positivity, tucatinib 300 mg twice a day and trastuzumab-anns every three weeks were added to the capecitabine regimen. The patient received one dose of the trastuzumab-anns, which led to an adverse reaction involving rigors, low blood pressure, and cold temperature. Brain MRI showed new and worsening enhancing lesions in the posterior fossa and diffuse pachymeningeal enhancement reflecting metastases. Given her current KPS of 50 and the progression of her disease on imaging, the patient was informed of her poor prognosis with an initial discussion of hospice care.

Due to the patient’s desire for additional treatment options, her young age, and the discovery of an actionable mutation in HER2 status, a decision was made to attempt sixth-line treatment with trastuzumab deruxtecan. In contrast to the lack of clinical response and numerous adverse effects associated with prior therapies, the patient’s response to trastuzumab deruxtecan is considered remarkable clinically and radiologically. During the time that the patient was on trastuzumab deruxtecan, the patient tolerated treatment well with the improvement of performance status from KPS 50 to KPS 90. Follow-up brain MRI at one month, three months, seven months, and 10 months after initiation of trastuzumab deruxtecan showed stable or minimally improving infratentorial cerebellar metastasis with no new metastatic lesions and continuously stable mild diffuse pachymeningeal enhancement. Given the patient’s excellent clinical response to trastuzumab deruxtecan, the patient had remained on trastuzumab deruxtecan for a total of 13 months, which gave her the opportunity to extend her lifespan with improvement in quality of life. At 13 months after initiation of trastuzumab deruxtecan, progression of leptomeningeal disease was noted on MRI, which showed leptomeningeal enhancement along the dorsal cervical cord at C2-C3 and the left L3 and S2 nerve roots. The patient was then transitioned to seventh-line treatment on pembrolizumab for one month, which was followed by eighth-line treatment on capecitabine, tucatinib, and trastuzumab for one month before her death in August 2023.

## Discussion

Recent paradigm shifts have placed a growing emphasis on precision medicine to provide targeted therapies for breast cancer patients based on their tumor characteristics. This patient had invasive lobular carcinoma of the breast, which is usually negative for HER2 with nearly 90% of patients testing negative for HER2 [6]. Although this patient’s initial pathology was negative for HER2, she was found to have HER2 positivity in her CSF and at a site of liver metastasis. Here, we discuss two possible hypotheses for the discrepancy of the HER2 prognostic marker in this patient: tumor heterogeneity and evolution of prognostic marker status.

Interestingly, there was a discrepancy in the biomarkers for this patient’s cancer from the time of initial diagnosis and liquid biopsy to the time of CSF sampling and liver biopsy. The patient’s initial pathology from her tissue biopsy in October 2017 and liquid biopsy obtained from blood sampling in March 2021 revealed HER2-negative cancer. A liquid biopsy is a test that monitors changes in circulating tumor DNA (ctDNA) to detect actionable gene alterations and predict response to targeted therapy [7-9]. Technologies for ctDNA detection are extremely sensitive and can detect alterations at a level as low as 0.01%-2% of genetic material [7]. However, as the clinical course of the patient continued, CSF sampling in January 2022 and subsequent liver biopsy in February 2022 were significant for HER2-positive cancer. CSF sampling is also a very sensitive technique, especially since this patient’s sample only contained five isolated tumor cells and of these cells, only one cell demonstrated HER2 amplification. CSF cytology is considered the gold standard for breast cancer leptomeningeal metastasis, but it also has a high false-negative rate (sensitivity: 45-67% of first lumbar puncture) due to small-volume samples and it often requires repeated lumbar punctures [10]. It is important to consider the possibility that this patient’s initial liquid biopsy may not have had the power to detect HER2 positivity or that over time, this patient’s cancer may have evolved to develop HER2 positivity, which was later detected via NGS of CSF.

Although it is common practice to categorize breast cancer based on prognostic markers, many types of breast cancer may exhibit tumor heterogeneity. Within a single tumor, there can be multiple subtypes that are due to varying states of differentiation and plasticity of cancer stem cells [1]. In addition, biopsies from geographically distinct areas have also been shown to exhibit subclonal diversification [2]. A biopsy might be subject to sampling bias in which a sample may not be representative of the entire tumor [3]. Given this information, it may be possible that the patient received an initial biopsy that represented an area of ER strong positivity (90%) and HER2 negativity (1+), which is what guided initial treatment with hormonal therapy rather than HER2-targeted treatments. Another interesting thing to note about this patient is that although her initial tissue biopsy demonstrated ER strong positivity (90%), her CSF analysis did not detect ER and her liver biopsy demonstrated ER with weak positivity (11%-50%). Of note, the change in ER predominance did not guide this particular patient’s treatment course.

Throughout the course of a patient’s treatment, it may also be possible for the prognostic markers to evolve. A retrospective study of 104 patients in Sweden showed that nearly 15% of patients had an alteration in HER2 status from the primary tumor to the relapse [4]. Another retrospective study of 110 patients from Greece showed that 18% of patients had a discordant expression of HER2 in the primary tumor and paired recurrences or metastases [5]. Growing evidence has suggested that the tumor microenvironment may be a driving force for clonal selection [11,12]. Another plausible explanation for the change in this patient’s prognostic markers could be that a change in the tumor microenvironment may have allowed for the proliferation of particular tumor subtypes, such as those with HER2 positivity. For this reason, it may be important to repeat biopsies for patients who have suboptimal responses to treatment to determine if tumor characteristics have evolved [4].

As the patient was nearing the end of available options for treatment, she was recommended to undergo lumbar puncture and NGS to further examine the presumed brain metastases. Leptomeningeal disease affects about 5% of patients with breast cancer and is associated with a poor prognosis with a median survival of about four months [13]. In this patient, Biocept analyses for NGS detected HER2 positivity, which led to further investigation and discovery of HER2-positive cells at a site of liver metastasis, which allowed the patient to receive treatment with HER2-targeted therapies. After the initial discovery of HER2 positivity, the patient began treatment with tucatinib, trastuzumab, and capecitabine, a regimen that was studied under the HER2CLIMB trial [14]. This trial showed that among patients with HER2-positive metastatic breast cancer with brain metastases, the addition of tucatinib to trastuzumab and capecitabine led to better progression-free survival at one year (24.9% vs. 0%) compared to placebo [14]. Tucatinib, along with other HER2 tyrosine kinase inhibitors, has been shown to effectively penetrate through the blood-brain barrier, which is especially useful in managing brain metastases [15]. However, this particular patient did not tolerate this regimen well due to adverse reactions and was therefore switched to a different regimen using trastuzumab deruxtecan.

Trastuzumab deruxtecan is a HER2-directed antibody and DNA topoisomerase I inhibitor conjugate that has been historically used as a treatment for patients with metastatic HER2-positive breast cancer [16]. It has been shown to have significant anti-tumor activity in pretreated patients with metastatic HER2-positive breast cancer [17]. Furthermore, trastuzumab deruxtecan was recently approved in 2022 for HER2-low (1+) metastatic breast cancer and has been associated with longer progression-free (9.9 months vs. 5.1 months) and overall survival (23.4 months vs. 16.8 months) compared to physician’s choice of chemotherapy [18]. Trastuzumab deruxtecan has also been shown to exhibit particular efficacy in the treatment of breast cancer brain metastases by being able to penetrate the blood-brain barrier [19]. The phase 2 TUXEDO-1 trial has shown that trastuzumab deruxtecan had a high intracranial response rate in patients with HER2-positive brain cancer and brain metastases with a best overall intracranial response rate of 73.3% [20]. It is also worth noting that since the time our patient received treatment, trastuzumab deruxtecan was approved for HER2-low (1+) metastatic breast cancer and so our patient would not have been previously approved for this treatment given her initial HER2-low status.

## Conclusions

Here, we reported the importance of subsequent tumor testing and the advantage of using NGS for identifying new targets and potential treatment options. It is therefore important to continue exploring a patient’s cancer throughout the disease course and continue following the pathology to open the doors for other opportunities for treatment. This demonstrates the potential for cancer to evolve and the benefit of serial determination of biomarkers to ensure the correct targeted therapies are provided to the patient.
